# A Rational Approach for the Production of Highly Soluble and Functional Sunflower Protein Hydrolysates

**DOI:** 10.3390/foods10030664

**Published:** 2021-03-19

**Authors:** Sophie Beaubier, Sara Albe-Slabi, Arnaud Aymes, Marine Bianeis, Olivier Galet, Romain Kapel

**Affiliations:** 1 Laboratoire Réactions et Génie des Procédés, Université de Lorraine, Unité Mixte de Recherche CNRS/Ministère (UMR) 7274, LRGP, F-54500 Vandœuvre-lès-Nancy, France; sophie.beaubier@univ-lorraine.fr (S.B.); sara.albe-slabi@univ-lorraine.fr (S.A.-S.); arnaud.aymes@univ-lorraine.fr (A.A.); 2 Avril SCA, 11 Rue de Monceau, F-75008 Paris, France; marine.bianeis@groupeavril.com (M.B.); olivier.galet@groupeavril.com (O.G.)

**Keywords:** enzymatic protein hydrolysis, plant protein, sunflower protein isolate, protein hydrolysate, solubility, functional food ingredient, enzymatic mechanism

## Abstract

Exploitation of plant proteins as an alternative to animal proteins currently presents an important challenge for food industries. In this contribution, total sunflower protein isolate from cold press meal was used as a starting material for the generation of highly soluble and functional hydrolysates that could be used in various food formulations. To do this, a rational and complete approach of controlled hydrolysis was implemented using the individual Alcalase and Prolyve enzymes. The method of stopping the hydrolysis reaction was also evaluated. The influence of operating conditions on hydrolysis kinetics and enzymatic mechanism was studied to identify the appropriate hydrolysis conditions. The gain of the solubility was then analyzed and compared to that of the initial proteins. Finally, the emulsifying and foaming properties (capacities and stabilities) of the resulting hydrolysates were also assessed. As a result, controlled enzymatic proteolysis significantly improved the sunflower protein solubility at neutral pH (twofold increase) and generated highly soluble hydrolysates. The limited proteolysis also maintained the good foam capacities and allowed an improvement in the initial foam stabilities and emulsifying capacities and stabilities of sunflower proteins. This contribution can greatly increase the value of sunflower meal and help in the development of sunflower protein products in the future.

## 1. Introduction

Exploiting new credible sources of plant proteins is gaining considerable attention. Proteins from sunflower meal have increasingly been studied to propose an alternative to proteins from animal origin [1,2]. Sunflower meal proteins are divided into two major fractions: globulins (helianthinins; 50–70%; 300–350 kDa; isoelectric point pI 4–6) and albumins (20–35%; 10–18 kDa; pI around 9) [1,3]. Amino acid composition of sunflower proteins is well-balanced (except for lysine) compared to World Health Organisation/Food and Agriculture Organisation/United Nations (WHO/FAO/UN) recommendation [4] and rich in sulfur-containing amino acids contrary to soy proteins. It is also reported that sunflower proteins have low contents of allergen factors and may exhibit interesting techno-functional properties such foaming and emulsifying [1,5]. Hence, production of sunflower protein isolates (protein content >90% on dry matter basis) might have a great potential in the food industry. However, several issues related to both plant source and technical processes (oil extraction, isolate production) currently limit the use of plant protein isolates in food. Another issue is related to undesirable organoleptic properties and/or a poor solubility compared to animal proteins.

Many processes for the production of sunflower protein isolates from solid meal have previously been reported. The most common one involves two main steps: a solid/liquid extraction in aqueous media at alkaline pH followed by a purification step performed mainly by acidic precipitation [5,6]. Alkaline extraction is widely implemented due to the solubility profile of sunflower proteins in water which exhibits minimal solubility around the isoelectric point of sunflower globulins (pH 4–6) [7]. These conventional processes usually result in an unsuitable dark green-colored powder which may also be accompanied by a decrease in nutritional and techno-functional quality [1,3,8]. Moreover, it has been reported that the acidic precipitation denatured the globulins and caused a high loss of the water-soluble albumins leading to a decrease of the global isolate solubility [7,9]. A recently published work of Albe-Slabi et al. [10] described the multicriteria optimization of the production of a total sunflower protein isolate from cold press meal. They reported the optimal extraction conditions (pH 7.3 and 0.3 mol·L^−1^ NaCl) and purification step by ultrafiltration. The solubility of this isolate was significantly improved using ultrafiltration process but remained particularly low at neutral pH (around 40%).

Enzymatic hydrolysis of proteins involves the action of proteases which catalyze peptide bonds hydrolysis at specific protein sequence portions (protease specificity) [11,12]. This results in a hydrolysate, which is a complex mixture of different protein fragments (peptides) of varying concentrations and molecular weights, free amino acids and may contain residual intact proteins. The hydrolysate composition is characterized with two main parameters: the protein conversion rate (Xp) and the mean size of the peptides produced (Naa). The Naa can be expressed by a mean number of amino acids composing the peptide of the hydrolysate and the Xp represents the ratio of protein hydrolyzed on the initial protein at a given time of the hydrolysis reaction [13]. The advancement of the hydrolysis reaction is commonly determined with the degree of hydrolysis (DH) which represents the ratio of peptide bonds cleaved on protein peptide bonds expressed in percentage [14]. 

It is well known that solubility, and thus techno-functional properties, are related to the hydrolysate composition [11,15,16,17]. The higher solubility of the hydrolysates compared to that of the initial proteins is mainly explained by the release of polar functions from the cleavage of peptide bonds. This is associated with a protein structure modification which exposes hydrophilicity functions (buried in the native structure) to the aqueous solvent [18]. Moreover, the release of large peptides can improve the functional properties of the proteins. At a given reaction advancement (DH), the hydrolysate composition (Xp and Naa) can vary according to the cleavage specificity of the applied protease and the enzymatic mechanism. However, at high reaction advancement (DH), the hydrolysates will end up being composed of small peptides, whatever the hydrolysis mechanism involved [12]. Small peptides are less effective in reducing interfacial tension and stabilizing the emulsions or foams formed [15]. Hence, extensive enzymatic hydrolysis (high DH) may be to the detriment of the techno-functional properties of proteins. An appropriate trade-off between the improvement of the solubility and the techno-functional properties may thus be difficult to identify. 

To date, many studies have reported the use of “limited” or “partial” proteolysis as a tool for the improvement of the functionalities [16,19]. In these classical approaches, only one chosen set of operating conditions is empirically applied for each enzyme and properties are then evaluated for the hydrolysates produced. The choice of enzyme used was usually not explained and the hydrolysates were characterized with the DH value only, mainly between 3% and 10% [20]. Also, the enzymatic mechanism is almost never analyzed in proteolysis studies. According to Linderstrom-Lang theory [21], there are two main mechanisms, the “zipper” mechanism and the “one-by-one” mechanism [12]. These mechanisms are associated to the exposure of the cleavage sites, i.e., to the protein structure, which can be controlled by the operating conditions (pH, temperature T, enzyme/substrate ratio (E/S)). Hence, the analysis of the enzymatic mechanism according to the protein/protease couple and the operating conditions applied seems essential to rationally improve the solubility and maintain the techno-functional properties of the initial proteins. 

In this contribution, a total sunflower protein isolate (SPI) extracted from cold press meal were enzymatically hydrolyzed. It was a continuation of a previous work where the optimal extraction conditions of these proteins have been highlighted (Albe-Slabi et al. [10]). The purpose of this present work was to control the implementation of enzymatic hydrolysis to improve the poor solubility of the SPI at neutral pH while at least maintaining the techno-functional properties. To do so, we described and followed a rational scientific strategy. The approach includes and discusses the choice of the enzymes, the analysis of the conditions of the reaction stops, the analysis of the enzymatic mechanism to identify the appropriate operating conditions and control the hydrolysate compositions, and eventually, the measurement and comparison of the functionalities (solubility, foaming and emulsifying properties).

## 2. Materials and Methods

### 2.1. Materials and Chemicals

The SPI was produced from a ground sunflower cold press meal provided by Olead (Pessac, France). The starting protein and fat content in the meal were 42.4% and 16.4% on dry matter basis, respectively. The enzyme Alcalase (2.4 L) from *Bacillus licheniformis* was purchased from Novozymes (Bagsvaerd, Denmark). The enzyme Prolyve (PAC 30 L) from *Aspergillus niger* were purchased from Soufflet Biotechnologies (Nogent-sur-seine, France). The proteases were food-grade. They were stored at 4 °C until it was used for the experimentations. A commercial soy protein hydrolysate (ISP 95 SA IP) from Solae (St. Louis, MO, USA) and a pea protein isolate (Nutralys^®^ F85M) from Roquette (Lestrem, France) were used. Sodium chloride and sodium hydroxide were purchased from VWR (Darmstadt, Germany) and hydrochloric acid was from Carlo Erba (Milan, Italy). All solvents (water and acetonitrile) were high-performance liquid chromatography (HPLC) grade and were purchased from Fisher Scientific (Hampton, VA, USA). The synthetic peptides used for the column calibration were purchased from GeneCust (Dudelange, Luxembourg). All other chemicals and reagents used were of analytical grade.

### 2.2. Production of Sunflower Protein Isolate (SPI)

The SPI was prepared as previously described by Albe-Slabi et al. [10]. Briefly, the sunflower proteins were extracted under optimal extraction condition (pH 7.3 and 0.3 mol·L^−1^ NaCl) and then purified by membrane processes including microfiltration and ultrafiltration. The solid/liquid extraction was implemented by mixing sunflower cold press meal with 0.3 mol·L^−1^ NaCl in 1:9 (solid: liquid) ratio. The mixture was stirred at 600 rpm. The temperature was kept at 20 °C and the pH was continuously adjusted to 7.3 by adding 1 mol·L^−1^ NaOH or HCl during the whole process, i.e., 60 min. The mixture was then centrifuged (15,000× *g*, 30 min, 20 °C) and the supernatant was clarified using Whatman filter paper. The clarified extract was then concentrated by microfiltration using a 0.22 μm cut-off by a volumetric reduction factor of 4. The retentate was washed with 2 diafiltration volumes of 0.2 mol·L^−1^ NaCl solution. The permeate was then concentrated by ultrafiltration using a 3 kDa cut-off by a volumetric reduction factor of 8 and was washed with 6 diafiltration volumes of 0.5 mol·L^−1^ NaCl. The pH of retentate was then adjusted to 9.0 using 1 mol·L^−1^ NaOH and it was washed with 4 diafiltration volumes of ultrapure water. The final retentate was eventually collected and freeze-dried. The obtained SPI had a dry matter content of 95.5%. The purity of this isolate was measured using the Kjeldahl method at 91.3% on dry matter basis (N × 5.6) consisting of 58% helianthinins and 42% albumins. The Kjeldahl method is described in Section 2.5.1.

### 2.3. Enzymatic Proteolysis of Sunflower Protein Isolate

The enzymatic hydrolysis of the SPI was carried out using the individual enzymes Alcalase or Prolyve. The proteolysis was conducted in a stirred and thermally controlled batch reactor of 200 mL under magnetic agitation. A constant initial SPI concentration was used (1%, *w*/*v*; in terms of protein content, N × 5.6). The SPI was dissolved in distilled water to prepare the protein substrate. Three different values of pH and E/S ratio were investigated for each protease. These values displayed in Table 1 were chosen according to the suppliers’ data. The temperature of hydrolysis reaction was constant at 50 °C for all reactions. The pH was maintained constant using an automatic titration system (902 Titrando, Metrohm Ltd., Herisau, Switzerland). The enzymatic hydrolysis reaction was for 2 h and the samples were recorded at given times of each reaction. Then, the reactions were stopped (as described in Section 2.4), and the samples were cooled down and stored at −20 °C.

### 2.4. Study of the Hydrolysis Reaction Stop

The proteolysis reactions were carried out under conditions where the enzymatic activities were optimal according to supplier’s data, i.e., E/S 1/0 and 50 °C for the two proteases, and pH 8.0 for Alcalase and pH 3.0 for Prolyve. The reactions were stopped when the reaction rates were high, i.e., after 10 min, (i) by heating during 15 min at 90 °C in a water bath or (ii) by shifting the pH at 3.0 for the sample with Alcalase by adding 1 mol·L^−1^ HCl or at 8.0 for the sample with Prolyve by adding 1 mol·L^−1^ NaOH (i.e., at 5 pH units from the optimal operating pH). Then, the optimal operating conditions of pH and temperature were implemented again during 30 min and a 1% (*w*/*v*; in terms of protein content, N × 5.6) substrate solution was added to validate the enzyme denaturation. The reactions were monitored with the automatic titration system (902 Titrando, Metrohm Ltd., Herisau, Switzerland). Samples were collected (i) before adding the protease, (ii) at the end of the first 10 min and (iii) after restarting the reaction and adding the fresh substrate for size-exclusion chromatography (SEC) analyses. 

### 2.5. Analytical Methods

#### 2.5.1. Kjeldahl Method

Total nitrogen content in the sample for protein purity and solubility calculations was determined in accordance with the official Kjeldahl method procedures described in the AOAC (Association of Official Analytical Collaboration) method 991.20 (1995, [22]). 1 mL of sample was prepared with 4 mL of 96% H_2_SO_4_ (*v*/*v*; Sigma-Aldrich, Saint-Louis, MO, USA) and approximately 10 mg of catalyst Cu-Se (AppliChem, Gatersleben, Germany). The mineralization step was then achieved in a digestion flask (Büchi SpeedDigester K−439, Rungis, France) at 450 °C during 150 min. The sample mixture was distilled in a Kjelflex K−360 (Büchi, Rungis, France) with 32% NaOH (*w*/*v*) afterward. After reaction with 3% boric acid (*w*/*v*) solution, the sample was titrated against 0.01 mol·L^−1^ HCl in a Titrino Plus 877 (Metrohm Ltd., Herisau, Switzerland). A blank was also analyzed with all reagents without the initial sample. To convert the total nitrogen content into a sunflower protein concentration, a conversion factor of N × 5.6 was applied according to Albe-Slabi et al. [10].

#### 2.5.2. Hydrolysate Characterization by Size-Exclusion Chromatography

The hydrolysis kinetics were monitored by SEC analyses according to the method developed by Beaubier et al. [13]. A Superdex peptide 10/300 GL column (fractionation range; 10 × 300 mm, GE Healthcare, USA) was used. 10 µL of sample were injected onto the column kept at 35 °C, connected to a Shimadzu model LC20 system (Shimadzu Corporation, Kyoto, Japan). An isocratic elution was used to separate the samples at 0.5 mL.min^−1^ with a water/acetonitrile/trifluoroacetic acid (TFA): 69.9/30/0.1 (*v*/*v*) solvent. The ultraviolet (UV) signal was recorded at 214 nm using a cell with an optical path of 0.5 cm. The column was calibrated with synthesized standard peptides eluted in the same conditions. The calibration equation was obtained by linear regression (Equation (1), R^2^ 0.87):

(1) $${\mathrm{MM}}_{x}{=\;10}^{-0{.06\times \mathrm{Tr}}_{x}+4.68}$$

where MM_x_ is the molar mass of the point ‘x’ of the chromatogram (g·mol^−1^) and Tr_x_ is the retention time of the point ‘x’ of the chromatogram (min).

The obtained hydrolysates from the SPI were also characterized according to the same method which allows quantifying simultaneously: the DH, the protein conversion rate (Xp) and the mean number of amino acids by peptide produced (Naa), [13]. The hydrolysate chromatograms were exported in Excel spreadsheets to determine the parameters. Peak area of dead volume eluents reflects the non-converted protein. The protein conversion rate is the proportion of the protein hydrolyzed at a given time and was thus determined by comparing the area at a given time to the initial area, as follows (Equation (2)):

(2) $${\mathrm{Xp}}_{\;}(\%)\;=\;(1-\frac{A}{A_{0}})\times 100$$

where A and A_0_ are respectively the final protein signal at the reaction stop and the protein signal before enzyme addition.

The peptide signal was used to determine the peptide molar weight distribution and the degree of hydrolysis of hydrolysates. The determination of the Naa value was based on the method of Bodin et al. [23], which propose to convert absorbance profiles of the peptide signal into concentration profiles by using the Beer–Lambert law. To do so, the mean molar mass of amino acid and the mean molar extinction coefficient by amino acid were calculated from the sequences of sunflower proteins in the proportion of the isolate studied, as Mw_aa_ = 113 g/mol and ε_aa_ = 938 M^−1^·cm^−1^, respectively.

Naa was thus calculated according to the following formula (Equation (3)):

(3) $$\mathrm{Naa}=\;\frac{n_{\mathrm{aa}}}{n_{p}}$$

where n_aa_ and n_p_ are respectively the molar quantity of amino acids and peptides in the hydrolysate, determined as (Equations (4) and (5)):

(4) $$n_{p}=\;\mathrm{Qv}\int \left(\frac{A_{x}}{\varepsilon_{x}l}\right)\;\mathrm{dt}$$

(5) $$n_{\mathrm{aa}}=\;\mathrm{Qv}\int \left({\mathrm{Cp}}_{x}{\;\overset{\text{¯}}{N}\mathrm{aa}}_{x}\right)\;\mathrm{dt}$$

with Q_v_, the elution flow rate and dt, a fraction of the elution time. A_x_ and $\varepsilon_{x}\;$are respectively the absorbance and the molar extinction coefficient for point ‘x’ of the hydrolysate chromatogram and l the path length of the light beam. Cp_x_ is the peptide concentration for each point ‘x’.

The mean amino acids number of the peptide mixture for each point ‘x’ (${\bar{N}\mathrm{aa}}_{x})\;$can be determined as the molar mass of the point ‘x’ of the chromatogram divided by the mean amino acid molar mass of the hydrolysate (determined from the protein hydrolyzed aminogram). The DH represents the percentage of peptide bonds cleaved compared to the initial number of peptide bonds of the protein and was achieved with Equation (6):

(6) $${\mathrm{DH}}_{t}\;\left(\%\right)=\;\frac{1}{\mathrm{Naa}}{\times \mathrm{Xp}}_{t\;}\left(\%\right)$$

#### 2.5.3. Techno-Functional Properties

##### Solubility

To determine the protein solubility, the SPI was dissolved in deionized water at a concentration of 5 g L^−1^ at room temperature. The pH was adjusted to a given value (2.0–9.0) by adding 0.1 mol·L^−1^ HCl or NaOH. The pH of the mixture was kept constant during 30 min under agitation and then centrifuged (1100× *g*; 10 min; 20 °C). The concentration of soluble proteins in the supernatant was measured using the Kjeldahl method as described in Section 2.5.1. The protein solubility was calculated as (Equation (7)):

(7) $$\mathrm{Solubility}\;\left(\%\right)\;=\;\frac{C_{x}{\times \;V}_{x}\;}{C_{i}{\times \;V}_{i}}\;\times 100$$

where C_i_ and C_x_ were the protein concentration in initial solution and in supernatant of the given solution (g·L^−1^), V_i_ and V_x_ were the volume of initial mixture and of mixture after pH adjustment (L), respectively.

The protein solubility of hydrolysates was determined after the hydrolysis reaction stop in the same way considering the initial protein concentration of the hydrolysis (1%, *w*/*v*; in terms of protein content, N × 5.6) and the collected sample volume.

##### Foaming Capacity and Foaming Stability

The foaming properties were assessed by the methods adapted from the reported works of Chabanon et al. [24]. 20 mL of the 1% (*w*/*v*, N × 5.6) protein solution was dissolved in deionized water and adjusted to pH 7.0. The solution was mixed at 10,000 rpm during 5 min using an Ultra-Turrax^®^ T25 digital homogenizer from IKA (Staufen im Breisgau, Germany). The foaming capacity was expressed (in %) as a ratio of the formed foam volume (mL) to the initial protein solution volume (mL). The foaming stability was calculated (in %) as the volume of the remaining foam after 120 min (room temperature) reported to the total volume of the solution at the given time. The foaming properties of a pea protein isolate and a soy protein hydrolysate assessed in the same way were used as references. 

##### Emulsifying Capacity and Emulsion Stability

The reported methods of Chabanon et al. [24] were applied with some modifications. 5 mL of the 1% (*w*/*v*, N × 5.6) protein solution was prepared in deionized water and the pH was adjusted to 7.0. The solution was then vigorously stirred at 10,000 rpm during 30 s using an Ultra-Turrax^®^ T25 digital homogenizer from IKA (Staufen im Breisgau, Germany). 2.5 mL of sunflower oil was added in the tube and homogenized for another 30 s at 10,000 rpm. After this time, 2.5 mL of sunflower oil was added again and stirred during 90 s at 10,000 rpm. The emulsion formed was transferred to a graduated tube and centrifuged at 1100 g for 5 min at room temperature. The emulsifying capacity (in %) was expressed as a ratio of the emulsion volume after centrifugation (mL) and before the centrifugation (mL). For emulsion stability measurement, the samples in the tubes were heated at 85 °C for 15 min. After cooling at room temperature, they were centrifuged again at 1100× *g* for 5 min. The emulsion stability was calculated (in %) as a ratio of the volume of emulsion after and before the heat treatment (mL). The emulsifying properties of a pea protein isolate and a soy protein hydrolysate assessed in the same way were used as references.

### 2.6. Statistical Analysis

All experiments were performed in triplicate and data were expressed as means (*n* = 3) ± SD (standard deviation). Statistical analysis was performed using the freeware R (3.4.1.) and the Analysis ToolPak of Excel (Microsoft Office, Redmond, DC, USA). Fisher’s test was first achieved, after testing normality and homogeneity of variance, with a confidence interval of 95% (*p*-value ≤ 0.05). Pairwise comparisons with Student’s test were then performed to study significant differences between the variables, with a significance level a = 0.05. Values were considered as significantly different if *p*-value ≤ 0.05. Results of statistical analysis are presented with letters and samples with common letter are not significantly different.

## 3. Results

### 3.1. Solubility and Functional Properties of Sunflower Protein Isolate

The protein solubility in an aqueous medium over a wide range of pH is one of the main quality criteria for food application of protein isolate and a prerequisite for the other techno-functional properties [1,5]. Good solubility promotes rapid and uniform dispersion of protein molecules, influencing their adsorption at interfaces (oil/water and gas/water) and therefore the formation of emulsion or foam [9].

**Figure 1:**
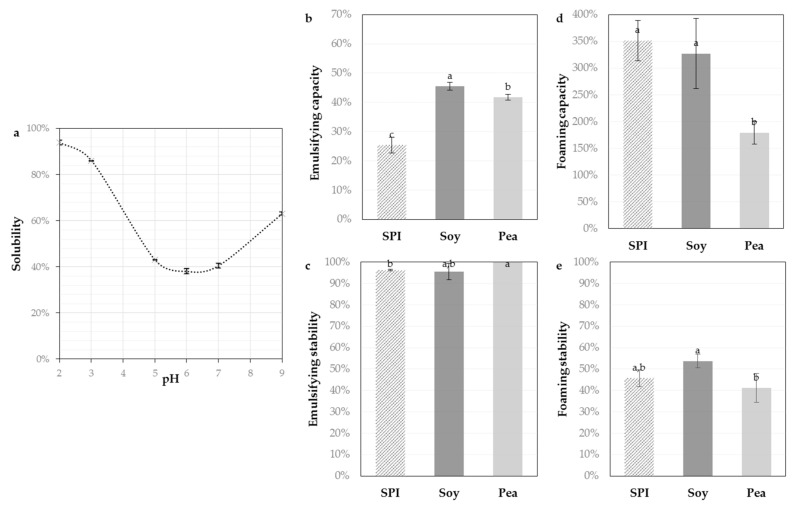
Solubility of sunflower protein isolate (SPI, 5 g L^−1^) as a function of pH ranging from 2.0 to 9.0 at 20 °C measured by the Kjeldahl method (**a**) and the techno-functional properties: emulsifying capacities (**b**), emulsifying stabilities (**c**), foaming capacities (**d**) and foaming stabilities (**e**) compared to a commercial pea isolate (Pea) and a hydrolysate of soy (Soy). Values are means (*n* = 3) and the error bars are the standard deviations. Samples with a common letter are not different (*p* > 0.05).

The solubility of the sunflower protein isolate (SPI) in the pH range 2.0–9.0 at 20 °C is depicted in Figure 1. The maximum solubility was observed under acidic (93.9% ± 1.1% at pH 2.0) and basic (63.1% ± 0.6% at pH 9.0) conditions. The minimum solubility was found around neutrality (40.6% ± 2.6% at pH 5.0–7.0). The solubility of sunflower proteins is related to their charge state and protein–protein interaction. The “U-shape” curve of their solubility curve is therefore explained by the isoelectric point which is between pH 4–6, [1,6]). In this pH range, the global net protein charge is null and so proteins tend to aggregate and precipitate. Outside isoelectric point range, proteins are charged. Electrostatic repulsion forces promote their solubility in water.

The techno-functional properties such emulsifying and foaming properties of the SPI were measured and compared to an isolate of pea and a partially hydrolyzed soy isolate. The emulsifying capacity (25% ± 3%) of the SPI was lower than those of references but the emulsifying stability (96%) was comparable and excellent. The foaming capacity (352% ± 38%) and foaming stability (46% ± 4%) of the SPI were good and significantly equivalent (even better) than those of references. This is consistent with the previous work of Albe-Slabi et al. [10] and the review of Gonzalez-Perez and Vereijken [1] on sunflower proteins and is explained by their high amount of hydrophobic amino acids.

Overall, the SPI solubility was improved by using an ultrafiltration process instead of an acidic precipitation [10], but it remained particularly low around pH 6.0 (+/−1). However, food formulations often take place at neutral pH, like meat products (sausages), pasta or bakery products (cakes, breads, desserts). Hence, it seems important to improve the SPI solubility in this pH range using limited enzymatic proteolysis, without altering the promising foaming and emulsifying properties 

### 3.2. Enzymatic Proteolysis of Sunflower Protein Isolate

#### 3.2.1. Protease and Reaction Stop Conditions Selection

The proteases used in the enzymatic hydrolysis process were usually chosen at random. In this work, the first step of the rational approach applied was to find the proteases which would be potentially the most effective for our purpose. To improve protein functionalities, endo-proteases with a low cleavage specificity are sought. These proteases hydrolyze the peptide bonds in the protein chains, releasing peptides of varying sizes, unlike the exo-proteases who hydrolyze at the end of the protein chains, generating small peptides and amino acids [15]. The proteases should be effective in the operating area of pH and temperature where the substrate proteins are the most soluble to promote exposure of the cleavage sites. In addition, the proteases must be food grade and have originated from microbial or plant (non-animal origin) to be used for food applications. For this reason, all gastrointestinal enzymes (pepsin, pancreatin, etc.) were avoided in this approach.

Sunflower proteins were the most soluble under acidic and basic conditions (Figure 1a). At higher temperature (50 °C), the SPI showed comparable solubility under acidic conditions but was almost totally soluble at pH 9.0 (data not shown). Thus, the enzymatic hydrolysis must take place within these ranges of operating conditions (pH 2.0–4.0 and 8.0–9.0 around 50 °C). Considering these observations, two proteases were chosen according to the recommendations of the suppliers: (i) the enzyme Alcalase (2.4 L; Novozymes) from *Bacillus licheniformis,* for the basic conditions (optimal recommended conditions of pH from 7.0 to 10.0 and 50–60 °C); and (ii) the enzyme Prolyve (PAC 30 L; Soufflet Biotechnologies) from *Aspergillus niger* for the acidic conditions (optimal recommended conditions of pH from 2.5 to 5.5 and 50–60 °C). Alcalase is widely applied in food research [20,25] but the use of Prolyve is rarely reported.

Batch proteolysis reactions need to be stopped by protease denaturation. Two techniques can be used for this purpose. The first one is raising temperature (above 90 °C for 3 to 15 min). The second one is pH shifting. For limited proteolysis, the chosen method may have a strong impact on the hydrolysate properties, mostly if a large proportion of protein remains intact. The pH or temperature treatment may indeed have different effect on protein structure and functionalities [1]. The protease denaturation method was usually not studied in proteolysis works. Here, both methods were evaluated with the selected proteases (Alcalase and Prolyve) in their recommended operating conditions, as described in Section 2.4. After stopping the reaction and adding fresh substrate, no reaction progress was followed again by the titration system. The SEC analyses also showed that the peptide fraction signal remained the same after stopping the reaction and 30 min after maintaining the optimal proteolysis conditions whatever the protease and the method used to stop the reaction (data not shown). This shows that the proteases were well denatured by both applied methods and proves irreversible reaction stops. To choose the most appropriate technique to stop the hydrolysis reactions of the SPI, its effect on the protein solubility measured at pH 6.0 and 20 °C was characterized. The SPI solutions were thus maintained under the optimal reaction conditions of both enzymes (Alcalase and Prolyve) for 10 min and then, the given methods for stopping reaction were carried out. To avoid modification of the solubility due to the protein hydrolysis, the enzyme was not added. As a result, no significant difference between protein solubility was found (average value of 41.3% ± 2.4%) whatever the protease and the method employed to stop the reaction. Both protease denaturation techniques can thus be used for the study of the limited hydrolysis of the SPI. However, the pH shifting results in salt addition which could decrease the protein purity of the hydrolysate. Hence, this study allowed choosing preferentially the method of temperature raising to reliably stop the hydrolysis reaction.

#### 3.2.2. Elucidation of Sunflower Protein Isolate Proteolysis Mechanism with Alcalase and Prolyve

As described by the Linderstrom-Lang theory [21], there are two main mechanisms, called the “zipper” mechanism and the “one-by-one” mechanism, based on the accessibility of the native proteins to the enzyme [12]. They are thus distinguished according to the ratio between the proteolysis steps: (i) the initial stage of denaturation and unmasking the peptide bonds of the protein (k1) and, (ii) the second stage of the subsequent hydrolysis of the unmasked peptide bonds that have become available (k2), [26]. In the “one-by-one” model, the first stage is limiting (k1 < k2), and the protein molecules are hydrolyzed one after another until the final products. Consequently, there are no peptides of intermediate sizes detected in this mechanism and the peptide profile remains similar during the reaction. In the “zipper” model, the first step in which the protein structure is denatured and unfolded is quicker than the step of the degradation to peptides (k1 > k2) and all protein molecules are hydrolyzed simultaneously. This results in a wide range of intermediate peptides of various sizes. Thus, the hydrolysates generated are qualitatively distinguished according to the enzymatic mechanism followed. The “one-by-one” mechanism generates hydrolysates composed of both residual intact proteins and small peptides, while it is possible to obtain a mixture of peptides of various size (long intermediate and small peptides) by “zipper-type” proteolysis [15]. As the hydrolysis mechanisms are associated to the protein structure, they can be influenced by the operating conditions of the hydrolysis process.

**Figure 2:**
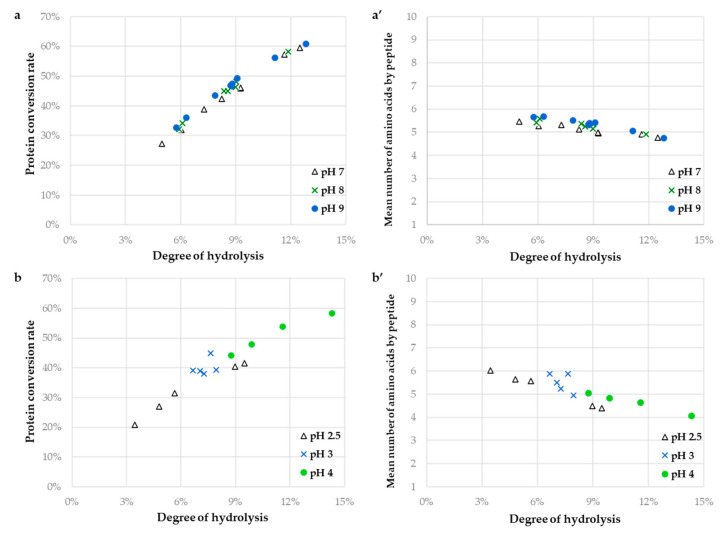
Protein conversion rate ((**a**,**b**); %) and mean number of amino acid by peptide (**a’**,**b’**) for the hydrolysis of the sunflower protein isolate (1%, *w*/*v*; in terms of protein content, N × 5.6) with Alcalase (**a**,**a’**) and with Prolyve (**b**,**b’**) as a function of the degree of hydrolysis as determined by size exclusion chromatography, for the different pH values of hydrolysis [Δ, pH 7.0; green x, pH 8.0; blue ○, pH 9.0; (**a**,**a’**); Δ, pH 2.5; blue x, pH 3.0; green ○, pH 4.0; (**b**,**b’**)] at 50 °C.

Figure 2 displays plots of the protein conversion rate (Xp, Figure 2a,b) and the average peptide size (Figure 2a’,b’) versus the DH for different enzymatic conditions (Table 1). These plots allow us to visualize the influence of the operating conditions on the reaction kinetics and/or on the mechanism of hydrolysis [13,27]. 

With Alcalase (Figure 2a,a’), Xp and Naa values vs. DH plots fell on the same curve whatever the operating conditions. A slight decrease in peptide size is observed from DH 4% to 13% where the average peptide size goes from around 6 to 5 amino acids. The Xp evolution as a function of the reaction advancement increased almost linearly (coefficient of determination of 0.978) reflecting a slow degradation of the intact proteins. This rather suggested that the “one-by-one” type mechanism of hydrolysis would be operating in the applied conditions [14]. It has previously been reported that in the investigated pH range of hydrolysis no denaturation of secondary structure of sunflower globulins and albumins was noted [1]. This could explain the observation of the same Xp profiles at all pH values. Hence, pH and E/S influenced DH kinetics but not the hydrolysis mechanism under the studied conditions. The hydrolysate compositions, solubility, and functionalities would probably only depend on the DH value (reaction advancement). This was consistent with the previous work of Chabanon et al. [24] on the hydrolysis of a rapeseed protein isolate.

For the hydrolysis with Prolyve (Figure 2b,b’), the behavior of the Xp and Naa values vs. DH plots were different according to the pH value. The Naa values were also around 5, but a more pronounced dispersion was observed (N_aa_ from 6 to 4 at pH 2.5, around 5 at pH 3.0 and from 5 to 4 at pH 4.0). The degradation of the proteins was also less linear in this case (coefficient of determination of 0.909) but was not quicker than that with Alcalase (around 50% at DH 10%). The proportion of the hydrolyzed proteins was dependent of the pH value (Xp from 21% to 42% at pH 2.5, from 38% to 45% at pH 3.0 and from 44% to 58% at pH 4.0, in the applied conditions). According to Butre et al. [27], the affinity of the protease for the hydrolysis of the intact proteins compared to that for the derived peptides can be estimated from the slope of the linear protein conversion rate. The affinity of Prolyve towards the proteins was 1.3 times higher at pH 2.5 than at pH 4.0 (for Alcalase case, no differences were observed depending on the pH value). This may be explained by the link between the susceptibility of the sunflower proteins to enzymatic hydrolysis and their structural stability. In this study, the hydrolysis rate was not related to the thermal stability because the same temperature was applied for all reactions (50 °C). But the SPI hydrolysis rate was closely related to the pH value with a decrease around the isoelectric point (pH 4–6 for the globulins), where the stability is highest. Hence, it is likely that the applied conditions in the case of Prolyve hydrolysis have an influence on both the enzymatic kinetics and mechanism. Moreover, both enzymatic mechanisms are likely to coexist depending on the pH value, with a slight prevalence of the “zipper” mechanism at more acidic pH. 

#### 3.2.3. Rational Choice of Operating Conditions and Degree of Hydrolysis (DH)

We highlighted that the hydrolysate composition depended only on the DH value with Alcalase in the applied conditions. Hence, hydrolysates with a wide range of DH should be generated to analyze the improvement of the solubility and techno-functional properties of the initial protein isolate. To improve the functionalities of the proteins, it is usually necessary to maintain a proportion of residual intact proteins in the hydrolysate and to produce peptides of substantial molecular weight. According to Figure 2, DH from 6% to 12% were thus produced with an increment of 2% (hydrolysates called A-DH6; A-DH8; A-DH10 and A-DH12), corresponding to proportions of intact proteins of 70% to 30%. As the operating conditions did not show any influence on the enzymatic mechanism, only one set of conditions was chosen in a range where the enzymatic activity was the highest and over a 2 h period (compatible with industrial applications). The operating conditions of the hydrolysis of the SPI with Alcalase were thus pH 8.0; E/S: 1/100 and 50 °C. This set differed from those previously reported for the SPI hydrolysis with Alcalase [28,29].

In the case of the hydrolysis with Prolyve, the previous analysis showed that the applied conditions had an influence on both the enzymatic kinetics and mechanism of hydrolysis. Hence, hydrolysates with a wide range of DH should also be generated, but different sets of operating conditions should also be implemented. According to Figure 2, DH from 4 to 12% were produced with an increment of 2%, corresponding to proportions of intact proteins of 75 to 45%. DH from 4 to 8% were produced at pH 2.5 (hydrolysates called P1-DH4; P1-DH6 and P1-DH8) and from 10 to 12% at pH 4.0 (hydrolysates called P2-DH10 and P2-DH12), all at 50 °C with E/S: 1/10.

### 3.3. SPI Hydrolysate Properties

#### 3.3.1. Solubility

**Figure 3:**
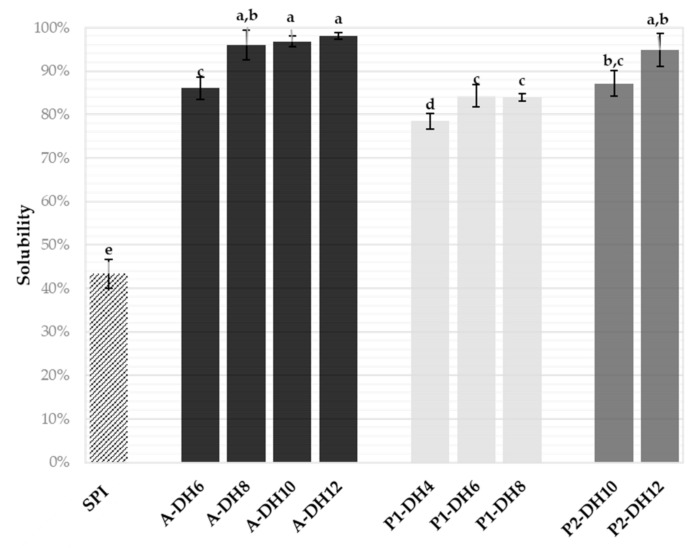
Solubilities of the sunflower protein isolate (SPI, 5 g·L^−1^) and of its hydrolysates generated with Alcalase (A) and Prolyve (P1 and P2) at different degree of hydrolysis (DH) measured by the Kjeldahl method at pH 6.0 and 20 °C. Values are means (*n* = 3) and the error bars are the standard deviations. Samples with a common letter are not different (*p* > 0.05).

The soluble nitrogen recovery of the nine chosen hydrolysates and the initial SPI was measured by the Kjeldahl method at neutral pH (6.0), i.e., where the SPI was the less soluble (Figure 3). As expected, the implementation of the rationally chosen hydrolysis significantly improved the initial solubility of the SPI (43.3% ± 3.4%) whatever the DH reached, and protease applied. The results showed a twofold increase even at low DH, with remarkably high values (78–98%). The hydrolysates obtained with Alcalase at DH 8%, 10% and 12%, and with Prolyve at pH 4.0 and DH 12% were almost totally soluble at pH 6.0, which is promising for potential use in many food applications. The solubility increase seemed related to the DH value increase. These results were consistent with previous works on improved plant protein solubility by enzymatic hydrolysis [24,30]. This was explained by the decrease of the molecular weight of the SPI and the surface hydrophobicity with the exposition to the aqueous solvent of polar groups initially buried in the native protein. 

On the other hand, the solubility was not significantly increased at DH higher than 8% for the hydrolysis condition with Alcalase, DH 6% for the hydrolysis with Prolyve at pH 2.5 and no significant differences were observed between the DH 10% and 12% hydrolysates with Prolyve at pH 4.0. This means that further hydrolysis than those mentioned was not necessary to improve the solubility. Hence, to observe differences between functionalities of the hydrolysates, the characterization of the foaming and emulsifying properties was only performed with the hydrolysates produced with Alcalase at DH 6% and 8% (significantly different solubility) and with Prolyve at pH 2.5 and DH 4% and at pH 4.0 and DH 10% (significantly different solubility).

#### 3.3.2. Techno-Functional Properties

**Figure 4:**
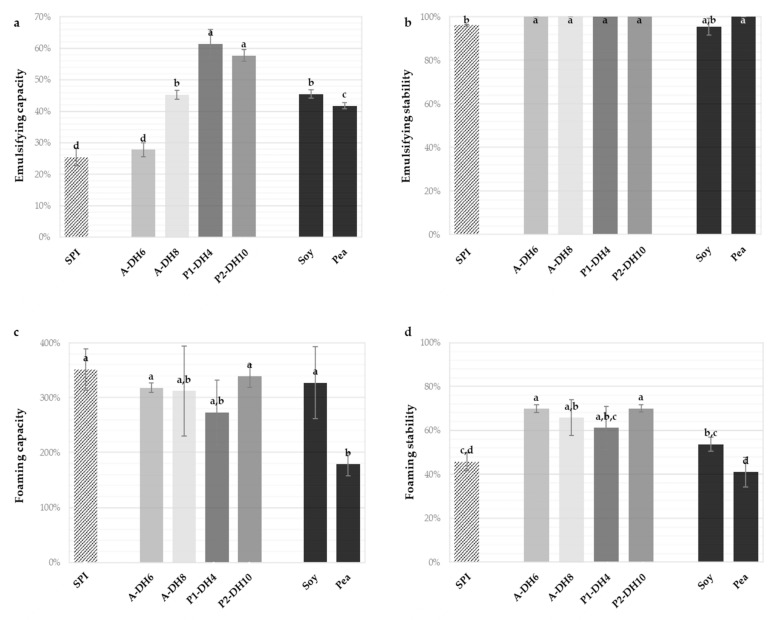
Emulsifying capacities (**a**), emulsifying stabilities (**b**), foaming capacities (**c**) and foaming stabilities after 120 min (**d**) of the initial sunflower protein isolate (SPI), the chosen hydrolysates and the commercial pea isolate and partially hydrolyzed soy isolate, measured at pH 7.0. Values are means (*n* = 3) and the error bars are the standard deviations. Samples with a common letter are not different (*p* > 0.05).

Figure 4 displays the emulsifying capacities and stabilities (Figure 4a,b) and the foaming capacities and stabilities (Figure 4c,d) of the initial SPI, the four selected hydrolysates, and the commercial isolate of pea and the partially hydrolyzed soy isolate. 

The hydrolysate obtained with Alcalase at DH 6% significantly maintained the emulsifying capacity of the SPI (around 28%). The three other hydrolysates improved the emulsifying capacities, particularly those produced with Prolyve (around 60%), which appeared to be superior to both references. This finding is interesting in view of the lower initial emulsifying capacity of SPI compared to the references. All hydrolysates of SPI were also characterized by improved emulsifying stability and presented totally stable emulsions after 120 min (100%). This is particularly notable because many studies have reported decreases in the emulsifying properties of plant protein hydrolysates compared to the initial isolate [24,31,32]. This phenomenon may be caused by the solubility improvement and release of large and hydrophobic peptides which favors fast diffusion and adsorption of the molecules at the interface and the reduction of the interfacial tension. To confirm that, the droplet size measurements and relevant microscopy analysis should be conducted in future work. All produced hydrolysates also allowed us to maintain the excellent foaming capacity of the SPI and improved its foaming stability. The foaming capacity (around 300%) and stability (around 70%) were equivalent for the four hydrolysates studied. Hence, the applied hydrolysis conditions or the DH reached, which may influence the hydrolysate composition, had no influence on these properties. The hydrolysates showed higher foaming stability than the references. This was noteworthy since enzymatic hydrolysis was also usually in detriment to the foaming stability [15,29,33,34]. Chabanon et al. [24] also reported an improvement of foaming stability measured in the same way with the hydrolysis of globulin-based isolate of rapeseed proteins, but with much lower values (33–47%). The foaming properties obtained were also superior to those previously reported for the hydrolysis of sunflower globulins with Alcalase [29]. Thus, the optimal hydrolysis conditions validated the objective of maintaining the promising foaming and emulsifying properties with the significant improvement of the solubility. Moreover, some techno-functional properties were also improved and comparable, or superior, to commercial references.

#### 3.3.3. Characterization of hydrolysis parameters of hydrolysates

**Figure 5:**
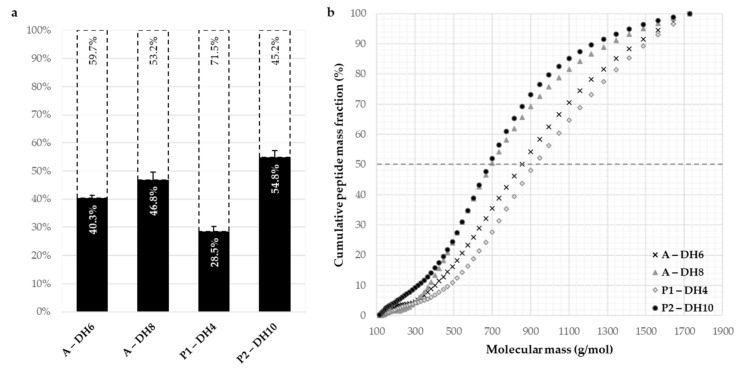
Protein conversion rates (dark blue histograms, %) and residual intact proteins (white histograms, %) of the chosen hydrolysates of the sunflower protein isolate (**a**) with Alcalase (A) and with Prolyve (P1 and P2) and the molecular weight distributions (**b**) determined by size exclusion chromatography. Chromatographic system: Superdex Peptide 10/300 GL column, detection at 214 nm, solvent: water/acetonitrile/TFA (69.9/30/0.1), flow rate of 0.5 mL·min^−1^.

The hydrolysate compositions of the four chosen hydrolysates are shown in Figure 5 where the proportions of hydrolyzed proteins and, therefore, the proportions of residual intact proteins, are graphically presented (Figure 5a). The cumulative peptide mass fraction of the hydrolysates analyzed by SEC is depicted as a function of the molecular mass in Figure 5b.

The obtained hydrolysates covered a large range of protein conversion rate from 28.5% to 54.8%. The repartitions of the molecular weights of the four hydrolysates had the same trends, but the median molecular weights were slightly distinguished according to the DH value of hydrolysate. The hydrolysate with Alcalase at DH 6% and that with Prolyve at DH 4% (the lowest DH) presented a median molecular weight of peptides around 900 g·mol^−1^ which corresponds to around 8 amino acids by peptide. For the two other hydrolysates with higher DH, the median molecular mass of peptides was lower, around 650 g·mol^−1^ (6 amino acids by peptide). 

A high amount of the SPI remained intact in the hydrolysates obtained. Furthermore, SEC analyses showed that the albumin fraction of the SPI was less hydrolyzed than the globulin fraction (data not shown). This can be explained by the high compactness of the albumin due to its low molecular weight (10–18 kDa), probably associated with a limitation of the accessibility of the protease cleavage sites. The fact that the albumins remained in solution, in parallel with the release of the polar functions due to the hydrolysis of the globulins, may explain the improvement in solubility and functional properties. Indeed, the albumins showed better functionalities than the globulins. This is explained by their low molecular weight that facilitates their adsorption to the interfaces and a higher amount of hydrophobic amino acids [35].

Except for the emulsifying capacity, all the functional properties analyzed were significantly equivalent for the four hydrolysates studied. Emulsifying capacity value of the hydrolysate produced with Prolyve at DH 4% was surprising compared to the others. Indeed, for the three other hydrolysates, the improvement in emulsifying capacity was associated with the decrease in the proportion of intact proteins (coefficient of determination of 0.976) and in the size of the peptides produced (coefficient of determination of 0.980). The hydrolysate P1-DH4 had the most important proportion of residual intact proteins (71.5%) and the biggest peptides and has shown the lowest solubility between hydrolysates (Figure 3). Hence, this improvement in the emulsifying capacity can be explained by the exposure of more hydrophobic amino acids to the solvent which can better interact with lipids to form an emulsion [11]. The hydrolysis of the same proteins by a given protease would result in comparable hydrophobic mixtures due to the cleavage specificity, particularly for endo-proteases. The hydrolysate also produced with Prolyve but at DH 10% showed equivalent emulsifying capacity. Therefore, the chosen protease Prolyve applied under the selected operating conditions would be likely to produce a peptide mixture of optimal hydrophobicity for this purpose. To validate this, the hydrophobic profiles of the hydrolysates were analyzed by reverse phase HPLC (RP-HPLC) and compared to the initial SPI (Figure S1). The hydrophobic peptide population was eluted from 37 to 53 min. The initial and intact SPI were eluted from 53 to 78 min. The peak peptides observed from 15 to 37 min represented small peptides that were less hydrophobic rather than high retention times. It was clearly observed that the intensity of the hydrophobic peptide fraction was much higher for both hydrolysates produced with Prolyve than those with Alcalase. This confirmed the aforementioned hypothesis.

As the hydrolysate composition had no influence on most of the properties tested, it would be judicious to select the optimal hydrolysis conditions on technical and economic criteria. The main technical and economic criteria of this process are the reaction time and the enzymatic cost (linked to the enzyme quantity). The enzymatic cost can be a limitation for the processing of plant protein isolates since the products have an intermediate added value. The hydrolysate obtained with Prolyve at DH 4% was produced the fastest but with a quantity of enzyme 10 times higher (E/S: 1/10, *w*/*w*) than the hydrolysates with Alcalase. As the decrease in the enzyme quantity would usually result in an increase in the reaction duration, a multicriteria optimization study could thus be interesting to carry out in this case [36].

## 4. Conclusions

This contribution describes a rational strategy for controlled implementation of enzymatic hydrolysis for the improvement of protein solubility and functionalities. For the first time, a complete approach was employed to study the influence of the operating conditions on the reaction kinetics and on the mechanism of hydrolysis of total sunflower protein isolate using Alcalase and Prolyve enzymes.

The results showed that the hydrolysis allowed the operating conditions to be accurately selected to generate hydrolysates with different compositions. The hydrolysates produced retained a significant proportion of intact proteins, sought to maintain techno-functional properties. Limited hydrolysis of sunflower proteins confirmed its effectiveness in improving their solubility. Also, the rational approach showed its potential to increase foaming and emulsifying stability, which is usually rarely reported with limited proteolysis. The described strategy made possible the identification and implementation of a controlled proteolysis to generate hydrolysates of sunflower proteins totally soluble at neutral pH, at the isoelectric point of proteins, that presented also good foaming and emulsifying properties. It makes them a potential ingredient to be incorporated in many food formulations and increases the value of defatted sunflower meal.

## Figures and Tables

**Table 1:** The operating conditions applied for the hydrolysis of the sunflower protein isolate with the individual proteases Alcalase or Prolyve for the kinetic studies.

Enzyme	pH			E/S Ratio (g Enzyme/g Substrate)			T (°C)
Alcalase (2.4 L)	7	8	9	1/10	1/50	1/100	50
Prolyve (PAC 30 L)	2.5	3	4				

## Data Availability

The data presented in this study are available on request from the corresponding author. The data are not publicly available due to the confidentiality of the project.

## Supplementary Materials

The following are available online at https://www.mdpi.com/2304-8158/10/3/664/s1, Figure S1: Chromatographic reverse-phase profiles of the initial sunflower protein isolate (dark lines) and the chosen hydrolysates of the sunflower protein isolate (pink lines): (a) Alcalase DH 6% (A-DH6), (b) Alcalase DH 8% (A-DH8), (c) Prolyve DH 4% (P1-DH4), (b) Prolyve DH 10% (P2-DH10).
