# Supplementary material for: A Rational Approach for the Production of Highly Soluble and Functional Sunflower Protein Hydrolysates
Source: Foods. 2021 Mar 19;10(3):664. doi: 10.3390/foods10030664 (PMC8003716; doi:10.3390/foods10030664)
Supplement: Supplementary file 1 [file foods-10-00664-s001.pdf]

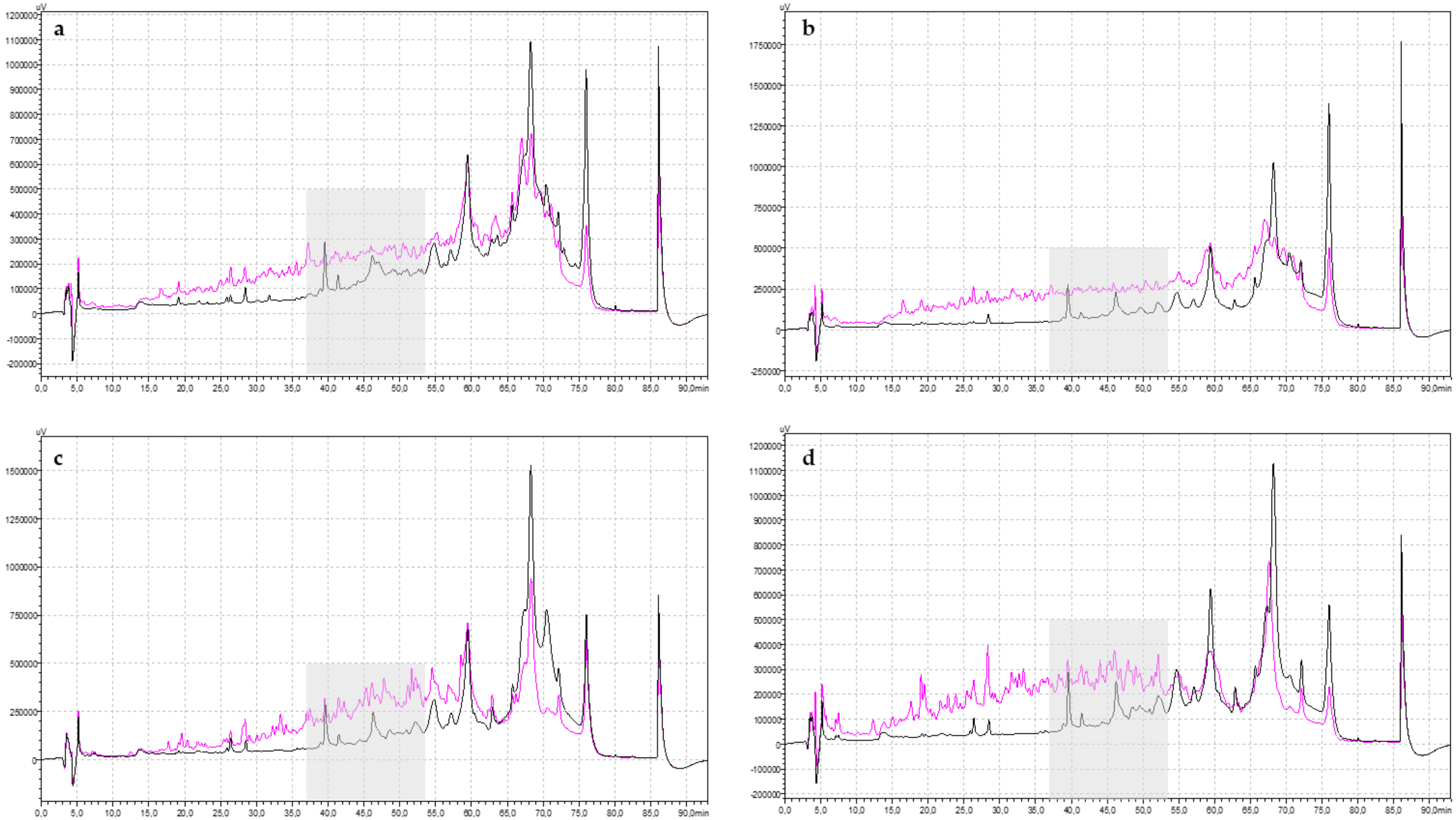

Figure S1. Chromatographic reverse-phase profiles of the initial sunflower protein isolate (dark lines) and the chosen hydrolysates of the sunflower protein isolate (pink lines): (**a**) Alcalase DH 6% (A-DH6), (**b**) Alcalase DH 8% (A-DH8), (**c**) Prolyve DH 4% (P1-DH4), (**b**) Prolyve DH 10% (P2-DH10).

Chromatographic system: C18 Prosphere (250 mm x 21 mm, 5 µm diameter beads) column, Shimadzu model LC20 system, detection at 214 nm, injection volume: 10 µL. Elution: gradient of solvent A (water/acetonitrile in 94.9/5 proportion (v/v) with 0.1 % TFA (v/v)) and solvent B (water/acetonitrile in 4.9/95 proportion (v/v) with 0.1 % TFA (v/v)), starting condition: 100 % A, a first slope applied to reach 28 % B/72 % A in 50 min, a second slope applied to reach 48 % B/52 % A in 20 min. The column was finally washed with 100 % B for 10 min and re-equilibrated in 100 % A for 15 min.
